# Nutrition, Healthcare Benefits and Phytochemical Properties of Cassava (*Manihot esculenta*) Leaves Sourced from Three Countries (Reunion, Guinea, and Costa Rica)

**DOI:** 10.3390/foods11142027

**Published:** 2022-07-08

**Authors:** Imane Boukhers, Frederic Boudard, Sylvie Morel, Adrien Servent, Karine Portet, Caroline Guzman, Manon Vitou, Joelle Kongolo, Alain Michel, Patrick Poucheret

**Affiliations:** 1 Qualisud, Univ Montpellier, Avignon Université, CIRAD, Institut Agro, IRD, Université de La Réunion, 34093 Montpellier, France; imane.boukhers@gmail.com (I.B.); frederic.boudard@umontpellier.fr (F.B.); adrien.servent@cirad.fr (A.S.); karine.portet@umontpellier.fr (K.P.); caroline.guzman@umontpellier.fr (C.G.); joelle.kongolo@univ-reunion.fr (J.K.); alain.michel@umontpellier.fr (A.M.); 2 CEFE, Laboratoire de Botanique, Phytochimie et Mycologie, CNRS-Université de Montpellier-EPHE-IRD, 34093 Montpellier, France; sylvie.morel@umontpellier.fr (S.M.); manon.vitou@umontpellier.fr (M.V.)

**Keywords:** cassava leaves, phenolic compounds, carotenoids, antioxidant activity, anti-inflammatory activity, nutrition, health

## Abstract

(1) Background: *Manihot esculenta*, cassava, is an essential food crop for human consumption in many parts of the world. Besides the wide use of its roots, cassava leaves have been used locally as green vegetables and for medicinal purposes. However, nutritional health data regarding cassava leaves is limited, therefore we investigated its composition and associated potential bioactivity interest for human health. (2) Methods: Cassava leaf bioactivity investigations focused on antioxidant properties (free radical scavenging) in association with immunomodulatory activities on inflammatory murine macrophages to measure the impact of cassava extract on the production of pro-inflammatory cytokines such as Interleukin-6, Tumor Necrosis Factor alpha, Monocyte Chemoattractant Protein-1, Prostaglandin-E2 and mediators such as nitric oxide. (3) Results: Antioxidant and immunomodulatory bioactivities were significant, with a concentration-dependent inhibition of cytokines production by inflammatory macrophages; (4) Conclusions: Taken together, our results tend to suggest that *Manihot esculenta* leaves might be underrated regarding the potential nutrition-health interest of this vegetal matrix for both human nutrition and prophylaxis of metabolic disease with underlying low grade inflammation status.

## 1. Introduction

The plant world is a major source of compounds with nutrition and health potential. Natural bioactive products are recognized for their beneficial effects in various human diseases. They are of great interest to researchers due to their broad structural diversity and wide range of biological activities [1].

Among them, cassava (*Manihot esculenta* Crantz) is known for its floury, starchy root, and is widely consumed in tropical regions of Africa, Asia and Latin America. According to the Food and Agriculture Organization of the United Nations, “cassava is an essential part of the diet of over half a billion individuals.” Global production totals over 250 million tons of fresh roots, which represents 32% of the world’s production of roots and tubers, second only to potatoes, which represent 45% of the total production [2]. Therefore, cassava is mainly grown as a root crop for human consumption as a source of carbohydrates. Nonetheless, in some regions, cassava leaves are consumed as green vegetables and are used in traditional medicines. They are also used as an ingredient in traditional sauces like saka-saka. Unlike roots, cassava leaves are an interesting source of nutrients such as proteins, vitamin A, vitamin C and fibers [3,4].

*Manihot esculenta* is a shrub that grows to about 5 m high and is part of the Euphorbiaceae family, considered one of the most complex and diverse angiosperm families, with about 300 genera and 8000 species [5]. As a function of the species studied, they are characterized by the presence of several secondary metabolites such as terpenoids, flavonoids and polyphenolic classes of compounds [6,7]. They contribute to various therapeutic properties, including anti-arthritic and anti-inflammatory activities [8] as well as inhibition of digestive enzymes including α-amylase, glucosidase and lipases. Such effects might be of interest for the prophylaxis and control of metabolic syndrome, type 2 diabetes and associated comorbidities, i.e., cardiovascular diseases [9]. The latter, according the World Health Organization (WHO) represents the leading cause of mortality in the world, with 17.7 million deaths each year, comprising 31% of total world mortality [10]. The most common risk factors for cardiovascular diseases include genetic factors, endothelial dysfunction, hyper-homocysteinemia, thrombosis, sedentary lifestyle, hypertension, hyperlipidemia, obesity, oxidative stress and inflammation [11]. Therefore, diet is a manageable risk factor that can play either a preventive or a first-line therapy role before any pharmacological treatment is needed. These observations explain the heightened scientific interest in the field of nutrition and health, with a focus on plant crop food secondary metabolites.

Polyphenolic compounds represent a family of organic molecules widely present in the plant kingdom. They are classified into different categories, including phenolic acids, flavonoids and non-flavonoids. They are associated with various properties, such as plant or fruit color, and are vegetal protective agents against external aggressors. These molecules constitute an area of interest for research on food bioactive compounds, as they are associated with various effects on human health [11]. More than 8000 polyphenols have been identified to date. They are found either in free aglycone form or in the form of glycoside esters. Numerous studies report anti-inflammatory and antioxidant properties that might contribute to preventive and/or therapeutic effects in metabolic and cardiovascular diseases [12,13]. Polyphenols antioxidant effects are based on different mechanisms, which vary according to molecular structure. Among the various mechanisms of action we can cite are: (i) scavenging free radicals such as OH• and NO• in particular, thanks to the polyhydroxyl and aromatic groups, or (ii) chelation of transition metals in the Fenton reactions, reducing free radical production from H_2_O_2_, e.g., curcumin and epigallocatechin gallate, or (iii) inhibition of NOX (NADPH Oxidase) production and/or xanthine oxidase, thereby reducing the production of reactive oxygen species (ROS), or (iv) lowering mitochondrial respiration attenuating the mitochondrial production of ROS such as O2• and H_2_O_2_ [11].

Some polyphenolic compounds also interact with cellular inflammation processes. EGCG (Epigallocatechin Gallate) inhibits NFκB (Nuclear Factor kappa B) activation through inhibition of IkBa (inhibitor of nuclear factor kappa B) degradation in human epithelial cells. It also downregulates the expression of iNOS (inducible Nitric Oxide Synthase) and NO (Nitric Oxide) production in macrophages. These effects are associated with a decreased expression of cytokines and pro-inflammatory mediators such as PGE-2 (Prostaglandin E2), IL-6 (Interleukin-6) and TNF-α (Tumor Necrosis Factor α) [14]. Additional in vitro studies revealed that EGCG, as well as polyphenols such as curcumin and kaempferol-3-O-sophoroside, inhibits the high mobility group box1 protein that interacts with nucleosomes, transcription factors and transcription-regulating histones, and plays a key role in inflammation [15]. Furthermore, in vivo and in vitro studies demonstrated that resveratrol could inhibit COX (Cycloxygenase), inactivate PPARγ (Peroxisome Proliferator-Activated Receptor Gamma) and induce eNOS (endothelial Nitric Oxide Synthase) in murine and rat macrophages [16]. Its cardioprotective effect was attributed mainly to its anti-inflammatory properties [17,18]. Polyphenols, such as quercetin, can activate the production of adiponectin, known for its anti-inflammatory effects. It also reduces the inflammatory state of adipose tissue by attenuating the expression of IL-6, IL-1β, IL-8, MCP-1, and TNF- α, preventing insulin resistance and improving dyslipidemia and hypertension [19,20].

Other secondary metabolites, the carotenoids, e.g., lycopene, are a family of compounds of more than 600 liposoluble plant pigments. Foods containing significant amounts of these compounds are considered to have beneficial effects on metabolic diseases, eye diseases and some types of cancers [16]. Carotenoids are natural pigments present in plants, fungi, algae and bacteria. They are not synthesized by the human body, and present a range of beneficial functions for health. Mechanistically, their main benefits can be explained by their antioxidant potential. However, some carotenoids may also act through specific mechanisms. For example, β-carotene has additional benefits due to its ability to be converted to vitamin A [21], while lutein, in addition to its ability to protect visual function, is thought to inhibit the initiation of inflammatory signaling pathways such as the Signal Transducer Activated and Activator of Transcription 3 (STAT3). Lutein treatment reduced the levels of nitric oxide (NO), tumor necrosis factor (TNF)-α, interleukin (IL)-6 prostaglandin (PGE-2) and monocyte chemotactic protein (MCP-1) in the aqueous humor of mice with endotoxin-induced uveitis. In addition, lutein depletion was observed in individuals with mild cognitive impairment and Alzheimer’s disease [22].

Although literature reports regarding the composition and bioactivity of cassava roots are available, the nutrition and health properties of cassava leaves are poorly described, especially regarding their antioxidant and immunomodulatory effects. Such knowledge might open the possibility for the intrinsic bioactivity of cassava leaves to be used for their pharmacological potential in the management of metabolic diseases such as metabolic syndrome and its co-morbidities (type 2 diabetes and cardiovascular diseases).

Therefore, the present study aimed to evaluate cassava leaves’ antioxidant and immunomodulatory activities by examining three samples harvested at the same stage of ripening from different countries where cassava root is widely consumed

## 2. Materials and Methods

### 2.1. Plant Material

Three different batches of *Manihot esculenta* leaves were collected from a 10-month plantation in the Reunion Island (CL.R), Costa Rica (CL.Cr), and Guinea (CL.G). For each sample, leaves were collected from 20 individuals planted in the same region, the identification was carried out by a botanist and a sample was kept in the laboratory of the Department of Pharmacology and Physiopathology at the University of Montpellier under code ME.CL032.

After collection, the leaves were carefully cleaned with distilled water and directly dried in a hot air oven at 45 °C for 48 h before being crushed and vacuum packed in sealed bags.

### 2.2. Extraction Procedure

The extraction of potentially bioactive compounds was performed as follows: briefly, 50 g of powder sample were dispersed in 200 mL of ethanol/water mix (80:20, *v*/*v*). The solution was sonicated with an ultrasonic bath Type vwr USC 300 TH for 30 min at 35 °C and then filtered on a membrane (Extract 1). The retentate was then subjected to a second extraction, identical to the first (Extract 2), and then a third with methanol/water (80:20, *v*/*v*) (Extract 3). The supernatants of the three extractions were combined and evaporated under a fume hood until dry.

### 2.3. Identification and Quantification of Phytochemicals

#### 2.3.1. Identification and Quantification of Carotenoids

Extraction of carotenoids from cassava leaves was performed three times on an ethanol/hexane mix (4:3, *v*/*v*) sample. The residue was separated from the liquid phase by filtration with a filter funnel (pore size #2). The organic phases of the three extractions were transferred to a separating funnel before the addition of 10 mL of NaCl to saturate the aqueous phase that was removed. The hexane phase was dried using anhydrous sodium sulfate and filtered before evaporation under vacuum. The carotenoid extracts were dissolved in 1 mL of 80:20 (*v*/*v*) mixture of methyl tert-butyl ether (MTBE) and methanol before being analyzed by HPLC.

Carotenoids were analyzed by HPLC using Agilent 1100 system (Agilent, Massy, France) with a diode array detector. Carotenoids were separated along a C30 column 250 × 4.6 mm i.d., 5 µm (YMC, Tokyo, Japan); the mobile phases were H_2_O as eluent A, methanol as eluent B, and MTBE (methyl-tert-butyl-ether) as eluent C with a gradient. The flow rate was fixed at 1 mL/min, the column temperature was set at 25 °C and the injection volume was 20 µL. The absorbance was measured at 470 and 450 nm. Chromatographic data and UV-Visible spectra were treated using the Agilent Chemstation plus software.

#### 2.3.2. Determination of Fibers and Ash

The determination of fibers and minerals was carried out according to the method of Van Soest [23] on dry matter of leaf powders. Fractionation of lignocellulosic biomass fibers was carried out by successive chemical extractions with neutral then acid detergents. For NDF, a neutral detergent solution (sodium lauryl sulfate, EDTA; pH 7) at boiling temperatures with a heat-stable α-amylase Termamyl^®^ was used to dis-solve the easily digested pectins and cell contents, leaving a fibrous residue NDF. For ADF, an acid detergent solution (cetyl trimethylammonium bromide and H_2_SO_4_) was used to dissolve hemicellulose. Finally, for ADL, a 3 h digestion was performed with 72% H_2_SO_4_.the dry insoluble fibers recovered by filtration were quantified by gravimetry.

### 2.4. Evaluation of the Bioactive Potential

#### 2.4.1. Antioxidant Activity

The antioxidant activity was evaluated in vitro by measuring the total polyphenol composition with the total polyphenol content (TPC) test, the free radical scavenging capacity with the DPPH (2,2-diphenyl 1-picrylhydrazyl) test, the antioxidant capacity with the ORAC test as well as the superoxide anion (SOA) immunolabeling test on living cells mitochondria.

##### TPC (Total Phenolic Content) Test

Total polyphenols assay was performed with Folin-Ciocalteu reagent according to the method of Morel et al. [24]. Leaf extracts of *Manihot esculenta* and of rosemary (internal reference) were prepared in DMSO at 4 mg/mL and then diluted in water to be tested at a concentration of 1 mg/mL. A calibration curve was generated on a concentration range of 1.56 to 75 μg/mL) of gallic acid. In a 96-well plate, 50 μL of extract, or 50 μL of gallic acid, and 50 μL of distilled water were distributed in triplicate. Then 50 μL of 10% Folin Ciocalteu reagent and 50 μL of sodium carbonate solution (1 M) were added. After 60 min in the dark the absorbance was measured on a microplate reader (Molecular Devices) at a wavelength of 650 nm. Results were expressed as milligrams of gallic acid equivalents (GAE) per gram of cassava plant leaf extract.

TPC calibration curve equation:  Y = 0.102X + 0.0353/R^2^ = 0.9991

##### DPPH (2,2-Diphenyl-1-picrylhydrazyl) Test

Antioxidant activity was evaluated using the DPPH assay according to the method of Morel et al. [24]. Extracts were solubilized in DMSO (4 mg/mL) before being diluted in absolute ethanol to reach a concentration of 1 mg/mL. A standard curve of Trolox was plotted (75, 50, 25, 12.5 µM). Ethanol was used as blank, ethanolic extract of *Rosmarinus officinalis* (0.2 mg/mL) and chlorogenic acid (0.01 mg/mL) were used as positive controls.

In a 96-well plate, 100 µL of positive control or extract were added in each well. The test was performed in triplicate for each extract. 75 µL of absolute ethanol and 25 µL of extemporaneously prepared DPPH solution (0.4 mg/mL) were introduced into each well. The plate was incubated for 30 min at room temperature and protected from light. The absorbance was read at 550 nm with a microplate reader (MDS Inc., Toronto, ON, Canada). Results were expressed as Trolox equivalents (TE µmoles per gram of dry extract) and as percentage of inhibition (% inhibition).

DPPH calibration curve equation:  Y = −0.0043X + 0.4575/R^2^ = 0.9986

##### ORAC (Oxygen Radical Absorbance Capacity) Assay

The ORAC (Oxygen Radical Absorbance Capacity) assays were performed in 96-well opaque polypropylene plates as previously described [24]. 

Samples were solubilized in DMSO at a concentration of 1 mg/mL before being diluted to 25 µg/mL using phosphate buffer at pH 7.4. On the 96-well microplate, 20 µL of Trolox solutions at 0.6, 25, 12.5, 25, 50 and 75 µM as standard curve, or chlorogenic acid (0.01 mg/mL), or ethanolic extract of rosemary (12.5 µg/mL) as a positive control, or the extracts at a concentration of 25 µg/mL, were applied. Then 100 µL of phosphate buffer and 100 µL of extemporaneously prepared fluorescein solution (0.1 µM in phosphate buffer) are added. The microplate was incubated at 37 °C for 10 min with shaking. The reaction was initiated with 50 µL of AAPH. Fluorescence was recorded at an excitation wavelength of 485 nm and an emission wavelength of 535 nm, for 70 min using a Tristar LB 941 microplate reader. Final ORAC values were calculated using a regression equation between Trolox concentration and area under the curve of decreasing fluorescein. Data are expressed as micromoles of Trolox equivalents per gram of dry extract.

ORAC calibration curve equation:  Y = 8229.46X + 24761.23/R^2^ = 0.9982

##### Determination of NO (Nitric Oxide) Scavenging

The cassava leaf sample was tested at different concentrations to determine its nitric oxide scavenging capacity, for this purpose the sample solutions were incubated with a volume ratio of 1:1 in 5 mM sodium nitroprusside solution diluted in HBSS for 2 h under a light source at room temperature. NO was then determined as nitrite by the Griess method using a standard curve of NaNO_2_ (1.56 to 100 µM), the reading was taken at 550 nm and the results were expressed as percent of NO scavenging.

##### Superoxide Anion Immunostaining Assay

Macrophage culture:

The macrophage cell line J774.A1 (ATCC, TIB67) was obtained from LGC Standards. Cells were cultured in RPMI 1640 GlutaMAX^®^ medium supplemented with streptomycin (100 µg/mL) and penicillin (100 units/mL), 10% inactivated fetal calf serum complete RPMI medium). Cells were incubated at 37 °C, 5% CO_2_ and 95% humidity.

Mitochondrial labeling:

Cells were seeded on a 24-well culture plate with complete RPMI medium. They were pretreated with various concentration of cassava leaf extract of 50, 25, 10 µg/mL for 4 h before being stimulated with LPS (100 ng/mL, E. coli 555B5) and murine interferon γ (10 ng/mL). They were then labeled with MitoTracker and MitoSOX probes following supplier instructions. The obtained images were processed with image J software for the measurement of probes fluorescence intensity. Results were expressed as MitoSOX/MitoTracker Ratio.

#### 2.4.2. Immunomodulatory Anti-Inflammatory Activity

##### Cell Viability by MTS/PMS Assay

To test cytotoxicity, 6.10^5^ cells/well were seeded in a 96-well culture plate in complete RPMI medium and incubated at 37 °C with different concentrations of extracts (25, 50, 75 and 100 μg/mL) for 20 h. After incubation, 20 μL/well of (3-(4,5-dimethylthiazol-2-yl)-5-(3-carboxymethoxyphenyl)-2-(4-sulfophenyl)-2H-tetrazolium), MTS, mixed with an electron coupling reagent, PMS in HBSS, were added. The plate was incubated for an additional 4 h and the absorbance at 490 nm was measured in a microplate reader (Molecular Devices) as previously described [24].

##### Productions of NO, PGE-2, IL-6, TNF-α and MCP-1

J774.A1 cells were seeded on a 24-well culture plate with complete RPMI medium. They were pretreated with various concentration of cassava leaf extract of 100, 75, 50 and 25 µg/mL for 4 h, stimulated with LPS (100 ng/mL) and interferon γ (10 ng/mL) and incubated for another 16–18 h at 37 °C. Supernatants were collected for nitrite, PGE-2, TNF-α, IL-6 and MCP-1 determination.

##### Determination of Nitrites (NO)

The presence of nitrite, a stable oxidized product of nitric oxide, was determined in the cell culture media as previously described [24]. Briefly, 100 μL of supernatant were combined with 100 μL of Griess reagent in a 96-well plate, incubated 10 min at room temperature. Nitrite concentration was determined by measuring absorbance at 550 nm using a NaNO_2_ standard curve (1.56 to 100 μM). Results were expressed as percentage of inhibition values.

##### IL-6 (Interleukin 6) Assay

IL-6 production by J774 cells was determined with the IL-6 ELISA-kit (Mouse IL6 ELISA; Thermo Fisher Scientific) after pretreatment with *Manihot esculenta* leaf extracts at a determined concentration range (25, 50, 75, 100 μg/mL) for 18 h. The cells were stimulated with 100 ng/mL LPS (E. coli, 555B5) and 10 ng/mL mouse IFN-γ for 4 h. IL-6 release in cell supernatants was tested according to the ELISA Kit instructions. The results for IL-6 as well as for all other pro-inflammatory cytokines are expressed as percentage of inhibition values.

##### TNF-α (Tumor Necrosis Factor Alpha) Assay

The tumor necrosis factor alpha (TNF-alpha) assay was performed according to the instructions contained in the kit-ELISA (TNF alpha Mouse Uncoated ELISA kit; Thermo Fisher Scientific). After pretreatment with the different concentrations of *Manihot esculenta* leaves for 3 h, the cells were stimulated with LPS 100 ng/mL (E. coli, 555B5) and mouse IFN-γ 10 ng/mL for 4 h. TNF-α release in cell supernatants was tested by sandwich enzyme-linked immunosorbent ELISA assay.

##### MCP-1 (Monocyte Chemoattractant Protein-1) Assay

Using the ELISA kit (Mouse CCL2 (MCP-1) (Thermo Fisher, Scientific), MCP-1 was detected in the cell culture supernatant 18 h after activation with LPS/IFN-γ. The supernatant was diluted 1:10 in EIA buffer to obtain a concentration of MCP-1 within the calibration range.

##### Prostaglandin Assay

The determination of prostaglandins E2 was performed by the competitive enzyme-linked immunosorbent assay (ELISA) on culture supernatants after pretreatment and subsequent activation of the cells with LPS/IFN-γ using the commercial Cayman PGE-2 ELISA KIT Monoclonal.

### 2.5. Statistical Analysis

All statistical analyses were performed using XLSTAT software version 2019.4.1 (Addinsoft, Paris, France). All data were reported as means ± standard deviation to the mean (SD) from three replicates of each experiment. Data were analyzed statistically using one-way analysis of variance (ANOVA) in order to determine significant differences (*p* < 0.05). Tukey’s multiple comparison method was used to further examine any significant difference between results.

## 3. Results

### 3.1. Phytochemicals Analysis

The phytochemical analysis of cassava leaf extracts is presented in the Table 1. Fiber levels were homogenous for each type of leaf. The same observations were made regarding major carotenoids, i.e., lutein and b-carotene. The lower amounts of carotenoids observed with the CL.G samples may be due to the known significant influence of cultivar and geographical region on cassava leaf composition. Our results are in global accordance with previous records at both qualitative and quantitative levels regarding the presence of β-carotene (40–80 mg/100 g dry matter) and lutein (8.6–77.21 mg/100 g dry matter) [25].

### 3.2. Antioxidant Bioactivity

Regarding phenolic compounds (Table 2), TPC results indicated that even if CL.R and CL.G presented no differences on this parameter, a significantly higher (20.94% higher TPC on average) total phenolic content in CL.Cr was recorded when compared to CL.R and CL.G samples. The three samples CL.R, CL.G and CL.Cr had, respectively, total polyphenol content values 2.29, 2.10 and 2.79 higher than the rosemary reference sample.

Results obtained with the DPPH and ORAC assays are presented in Table 2. It is interesting to note that, in spite of the phytochemical analysis that demonstrated a higher polyphenol content in CL.Cr when compared to the other samples, no statistical difference was recorded between the three types of cassava leaves for each type of assay. All three samples were, on average, 2.69 less bioactive than the Rosemary reference.

**Figure 1:**
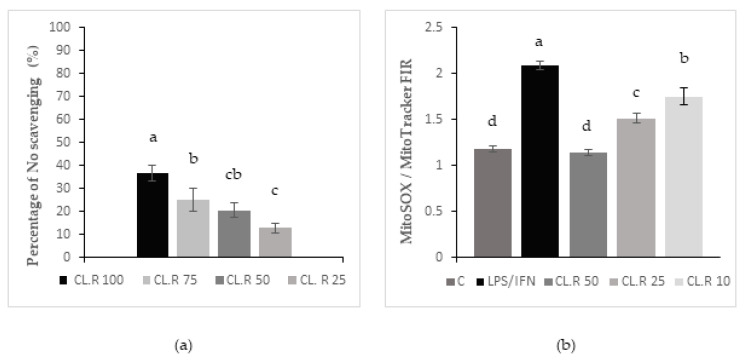
(**a**) Nitric oxide scavenging effect of *Manihot esculenta* leaves at different concentrations (100, 75, 50 and 25 µg/mL), values are expressed as percentage of NO scavenging plus or minus SD (*n* = 3) (a, b and c; *p* < 0.05). (**b**) Effect of *Manihot esculenta* leaves at different concentrations (100, 50, 25 and 10 µg/mL) on J 774 macrophages cells mitochondria versus control cells (C) and stimulated cells (LPS/IFN). Values are expressed as MitoSox/MitoTracker fluorescence intensity ratio (FIR) plus or minus SD (*n* = 3) (a, b, c and d; *p* < 0.05).

Nitric oxide (NO) scavenging results are presented in Figure 1a. The graph represents the levels of NO scavenging potential as a function of increasing concentrations of *Manihot esculenta* leaf CL.R extract concentrations from 100 to 25 µg/mL. Statistical differences were recorded between the 100, 75 and 25 µg/mL concentration. The 50 µg/mL concentration produced results that were similar to the 75 and 25 µg/mL concentrations, although the absolute value was roughly midway between the two. Nonetheless these results indicate that increasing concentrations of *Manihot esculenta* leaf extract were associated with an increased level of NO scavenging capacity. This observation indicated a dose-concentration scavenging bioactivity of *Manihot esculenta* leaf extract on the pro-inflammatory free radical nitric oxide generated by stimulated macrophage cells.

Results obtained with mitochondrial labeling are presented on Figure 1b. The assay presented was performed on *Manihot esculenta* leaf CL.R samples as it represents the effects obtained on the three types of leaf extracts. The graph represents the level of superoxide anion (SOA) production per mass of mitochondria in stimulated macrophages pre-treated with *Manihot esculenta* leaf extracts at various concentrations. All concentrations tested significantly inhibited superoxide anion production when compared to untreated stimulated macrophages. In addition, a dose-concentration effect was recorded with the higher concentration (50 µg/mL) of *Manihot esculenta* leaf extracts inhibiting SOA production back to unstimulated control levels.

### 3.3. Immunomodulatory Anti-Inflammatory Bioactivity

Immunomodulatory bioactivity assays presented were performed on *Manihot esculenta* leaf CL.R samples as they represent the effects obtained on the three types of leaf extracts.

Cell exposure to *Manihot esculenta* leaf extracts did not alter macrophages viability, thereby allowing the exploration of their anti-inflammatory activity without adverse influence (data not shown).

**Figure 2:**
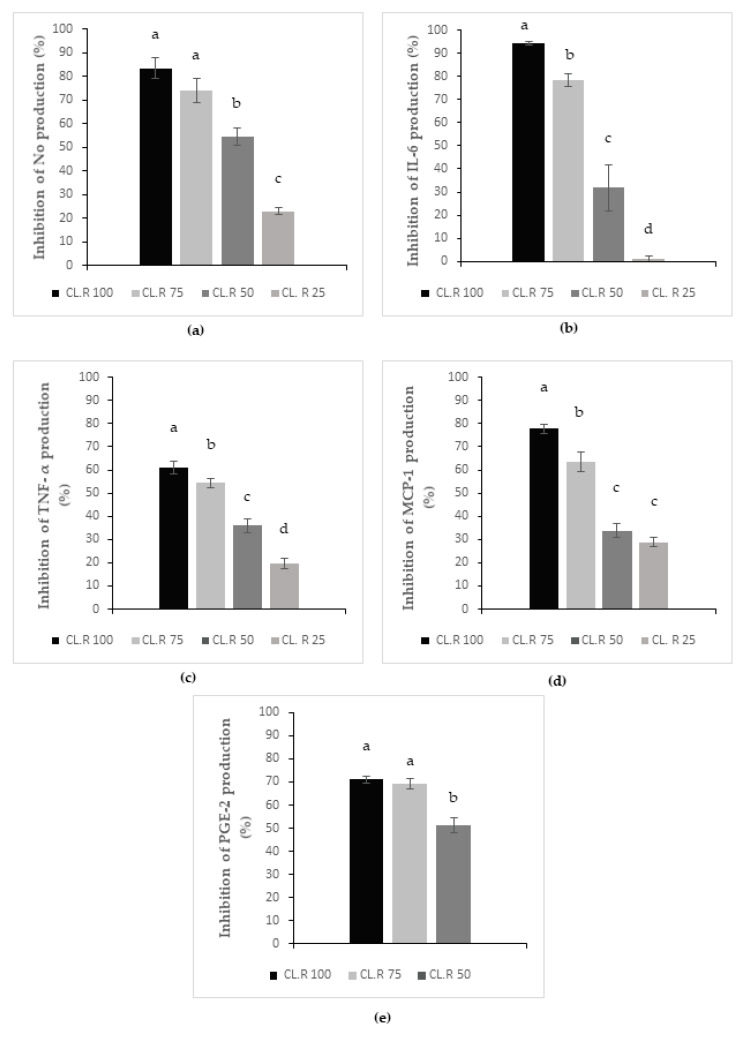
Effect of *Manihot esculenta* leaves at different concentrations (100, 75, 50, 25 µg/mL) on NO production (**a**) as well as on the production of pro-inflammatory cytokines: IL-6 (**b**), TNF-α (**c**), MCP-1 (**d**), and PGE-2 (**e**) by inflammatory J 774 macrophages. Values measured by Elisa are expressed as the mean of the percentage of inhibition plus or minus the standard deviation (*n* = 3) (a, b, c, and d; *p* < 0.05).

Nitric oxide (NO) production results are presented in Figure 2a. The graph represents the levels of inhibition of NO production as a function of increasing concentrations of *Manihot esculenta* leaf CL.R extract concentrations from 100 to 25 µg/mL. Concentrations of 100 and 75 µg/mL induced the highest levels of inhibition (over 70%) with no statistical differences between the two concentrations. However, statistical differences were recorded between the 75 µg/mL, 50 and 25 µg/mL concentrations. Therefore, it could be demonstrated that increasing concentrations of *Manihot esculenta* leaf extract were associated with an increased level of inhibition of NO production. This observation indicated a dose–concentration inhibitory bioactivity of *Manihot esculenta* leaf extract on the pro-inflammatory free radical nitric oxide production by stimulated macrophage cells.

Interleukin-6 (IL-6) production results are presented in Figure 2b. The graph represents the levels of inhibition of IL-6 production as a function of increasing concentrations of *Manihot esculenta* leaf CL.R extract concentrations from 100 to 25 µg/mL. Statistical differences were recorded between all four (100, 75, 50 and 25 µg/mL) concentrations. Therefore, it could be demonstrated that increasing concentrations of *Manihot esculenta* leaf extract were associated with an increased level of inhibition of IL-6 production. This observation indicated a dose-concentration inhibitory bioactivity of *Manihot esculenta* leaf extracts on the production, by stimulated macrophage cells, of interleukin-6; a pro-inflammatory cytokine involved in the acute phase of inflammatory processes.

Monocyte Chemoattractant Protein-1 (MCP-1) production results are presented in Figure 2d. The graph represents the levels of inhibition of MCP-1 production as a function of increasing concentrations of *Manihot esculenta* leaf CL.R extract concentrations from 100 to 25 µg/mL. Statistical differences were recorded between the first three concentrations 100, 75 and 50 µg/mL concentrations (the last concentration 25 µg/mL was not statistically different from concentration of 50 µg/mL). Nonetheless it could be demonstrated that increasing concentrations of *Manihot esculenta* leaf extract were associated with an increased level of inhibition of NO production. This observation indicated a dose-concentration inhibitory bioactivity of *Manihot esculenta* leaf extract on the macrophage production of MCP-1, a key chemokine for the regulation of monocytes/macrophages migration/infiltration processes.

Tumor necrosis alpha (TNF-α) production results are presented in Figure 2c. The graph represents the levels of inhibition of TNF-α production as a function of increasing concentrations of *Manihot esculenta* leaf CL.R extract concentrations from 100 to 25 µg/mL. Statistical differences were recorded between all four 100, 75, 50 and 25 µg/mL concentrations. Therefore, it could be demonstrated that increasing concentrations of *Manihot esculenta* leaf extract were associated with an increased level of inhibition of TNF-α production. This observation indicated a dose-concentration inhibitory bioactivity of *Manihot esculenta* leaf extract on the production, by stimulated macrophage cells, of TNF-α pro-inflammatory cytokine involved in the acute phase and systemic inflammation processes.

Prostaglandin E2 (PGE-2), production results are presented in Figure 2e. The graph represents the levels of inhibition of PGE-2 production as a function of increasing concentrations of *Manihot esculenta* leaf CL.R extract concentrations from 100 to 25 µg/mL. Statistical differences were recorded between 100 or 75 µg/mL and 50 µg/mL concentrations but not between 100 or 75 µg/mL. In addition, 25 µg/mL did not generate any inhibition (Data not shown). These data tend to suggest that increasing concentrations of *Manihot esculenta* leaf extract might be associated with an increased inhibition of PGE-2 production; a major prostanoid involved in inflammatory processes homeostasis. Based on the two concentrations 100 (or 75) mg/mL and 50 mg/mL and considering the absence of effect for 25 mg/mL concentration, the inhibitory bioactivity of *Manihot esculenta* leaf extract on the production of PGE-2, by stimulated macrophage cells, may possibly be considered as dose dependent.

## 4. Discussion

The present study was realized to explore *Manihot esculenta* (cassava) leaves’ bioactivity to contribute to a better knowledge of this food crop’s nutrition and health benefits. Indeed, in addition to its roots, cassava leaves are available to be used as green vegetables for human consumption, as is already the case in some regions such as South America, southeast Asia and Indonesia [26,27]. To date, cassava leaf bioactivity, as relates to antioxidant and immunomodulatory activities are, to our knowledge, poorly described.

Therefore, we investigated cassava’s global bioactive content, specifically polyphenols and carotenoids, in relation to its antioxidant and anti-inflammatory potential, with the perspective of a putative use in the management of metabolic diseases associated with oxidative stress and low-grade inflammation.

*Manihot esculenta* Crantz is an important staple food crop worldwide [28]. Its root represents, by itself, a major agro-industrial sector, leaving cassava leaves as a low value byproduct. Nonetheless, recent reports explored *Manihot esculenta* leaf composition and its associated putative biological interest [29,30]. Our results are in agreement with these reports, with TPC and carotenoid contents similar to literature data, providing support for our observations on bioactivity. For this reason, cassava leaves were recognized as a good source of macronutrients and micronutrients. Regarding macronutrients, cassava leaves contain significant amounts of proteins (up to 38.1%), essential amino acids (methionine and phenylalanine) and dietary fibers. Micronutrients include vitamins, minerals and secondary metabolites. These observations underline their global nutritional interest [29,30,31]. Cassava leaves may provide phenolic compounds and carotenoids [25,32]. Total phenolic compounds, including phenolic acids (e.g., gallic acid, syringic acid, hydroxybenzoic acid, etc.), flavonoids (e.g., rutin, apigenin, kaempferol, etc.) and anthocyanins are believed to contribute to cassava’s biological properties. In addition, this composition may vary significantly as a function of the cultivar, growth conditions, plant age and maturity, leaf position and post-harvest processing [25,33,34]. Carotenoids, such as beta-carotene and lutein, in addition to their nutritional value, are also recognized as bioactive compounds. Beta-carotene biological interest comes from its bioconversion in vitamin A. Lutein demonstrated various positive health effects in pathophysiological conditions such as age-related macular degeneration, cardiovascular diseases and metabolic syndrome [35,36].

Modulation of Radical Oxygen Species (ROS) production in the human body to support cells redox homeostasis is recognized as a major therapeutic goal for various pathologies, and more specifically, for non-communicable diseases such metabolic syndrome, obesity, type 2 diabetes and hypertension, as well as for some forms of cancer and neurodegenerative processes [33,37]. Our results, with rosemary as the internal assay validation control, demonstrated an antioxidant capacity of *Manihot esculenta* leaves consistent with previous reports [38]. Average cassava leaf total phenolic content was in the high range when compared to the literature [39,40]. The DPPH assay mimics free radicals’ production to assess the scavenging capacity of a test compound or extract based on its ability to provide a hydrogen atom. Cassava leaf extracts scavenged DPPH radicals and inhibited the oxidative insult by an average of 72.31%. These data were consolidated by ORAC measures, often used to assess food antioxidant capacity. The ORAC test monitors the scavenging of free radicals such as peroxyls predominantly associated to lipid peroxidation. In our case, the average ORAC antioxidant capacity reached an average of 3073.84 mmol/TE/g. As a comparison, beet green, green lettuce, spinach and broccoli were reported with ORAC values of 2442, 1532, 734 and 335, respectively [33]. Indeed, phenolic compounds are plants’ secondary metabolites with direct antioxidant effects as electrons donors, and in some case, they may also stimulate cells’ endogenous antioxidant defense systems [41,42]. The significant antioxidant, and more specifically, anti-peroxyl activity recorded in our assay, is in accordance with the literature. Indeed, Tsumbu et al. identified *Manihot esculenta* leaf extracts as having the higher level of protection against lipoperoxidation when compared to other selected green sub-Saharan vegetables [33].

Nitric oxide (NO) is a free radical involved in various functions in several organs, i.e., vasodilation in the cardiovascular system. It is also a mediator of oxidative stress and a pro-inflammatory mediator of both acute and chronic inflammation. Mitochondria’s main functions are cellular respiration and energy production associated with ROS generation. Mitochondrial SOA was demonstrated to be produced concomitantly with NO. SOA may react with NO to generate powerful oxidant molecules, i.e., peroxynitrite. In pathophysiological conditions, this would lead to increased production of both ROS, ultimately compromising mitochondrial functions and integrity [40] associated with major oxidative stress. Such a process is highly detrimental to mitochondria, as well as to cells and tissues whose structure and functions are negatively altered. Our results showed a concentration-dependent NO scavenging effect of *Manihot esculenta* leaf extracts as well as a concentration-dependent inhibition of its production by stimulated macrophage cells. In addition, the same extracts demonstrated a concentration-dependent inhibition of mitochondrial SOA overproduction in stimulated inflammatory macrophages. The combined effects of *Manihot esculenta* leaf extracts on NO and SOA production may not only decrease the exposure of cells and mitochondria to excessive amounts of harmful ROS, but also limit the amplification loop of peroxynitrite production. Such an effect would be of interest in avoiding mitochondria dynamic disturbances, bioenergetic failure, and ultimately, cell death [40].

Taken together, our data therefore suggest that *Manihot esculenta* leaf extracts offer protection against the deleterious effects of highly reactive oxygen species overexposure. Such extracts may contribute to the prevention of ROS-related mitochondrial, cell and tissue structural damages and dysfunction. This bioactivity may be associated not only to phenolic, flavonoid and carotenoid compounds themselves, but also to their potential synergistic pharmacological actions underlying their antioxidant mechanism of action [29]. Therefore, cassava leaf consumption might be of beneficial interest as part of a healthy diet, as well as in the prophylaxis of numerous pathophysiological conditions [31,33].

Inflammation processes and their associated production of inflammatory cytokines are normal physiological non-specific immune responses to combat organism assault. They are also recognized as important determinants in many human pathological conditions, and more specifically, non-communicable diseases such as metabolic syndrome, obesity, type 2 diabetes, cardiovascular diseases, oncogenesis and neurodegenerative diseases. In this context, phenolic compounds from vegetal biodiversity sources were reported as anti-inflammatory compounds both in vitro and in vivo [16]. Their molecular mechanism of action may involve modulation of various signaling pathways and molecular targets such as NF-kB (Nuclear Factor Kappa-light-chain-enhancer of activated B cells), MAPk (Mitogen-Activated Protein kinase), PI3K/Akt (Phosphatidylinositide 3-kinase/Protein kinase B), PLA2 enzyme (Phospholipase A2) and cyclooxygenases (COXs), leading to a reduction of prostaglandin production. In addition, polyphenols might regulate endogenous anti-inflammatory enzymes such as catalase, superoxide dismutase (SOD) and glutathione peroxidase [16]. In a similar way, clinical trials demonstrated that carotenoids’ anti-inflammatory properties would be expressed through the modulation of NF-kB and MAPk pathways, and their actions may be synergistic with polyphenols [42]. To explore the anti-inflammatory and immunomodulatory potential of cassava leaves, we exposed stimulated macrophages to cassava extracts and monitored various major inflammation biomarkers, i.e., the free radicals associated with inflammation nitric oxide (NO) production and scavenging, Prostaglandin-E2 (PGE-2), Interleukin-6 (IL-6) and Tumor necrosis factor-alpha (TNF-α) involved in homeostasis and the acute phases of inflammation and Monocyte Chemoattractant Protein-1 (MCP-1) involved in the cellular aspect of the inflammatory response. Cassava leaf extracts globally induced a dose-dependent inhibition of the inflammatory response, as suggested by the reduction in production levels of all biomarkers. These results are in accordance with polyphenol and carotenoids properties reported in the literature. To our knowledge, this is the first time that *Manihot esculenta* leaves are reported to demonstrate these bioactivities in vitro.

In addition, it also should be noted that our original results on *Manihot esculenta* leaves are also in agreement with an in vivo report mentioning the inhibition of a chemically induced acute inflammation in rodents after oral and topical administration of cassava leaf extract [43,44]. This point is of significant interest, as it strongly suggests that, in the case of cassava leaves, the intrinsic properties of the vegetal matrix, studied in vitro, may be transferable in vivo. This hypothesis raises several interesting research questions regarding the nature of the combination of bioactive compounds responsible for this health benefit. Furthermore, the knowledge that pain was alleviated concomitantly to inflammation in the in vivo report, combined with our data, suggests a possible partial explanation of the mechanisms of action of these effects. The PLA2/COX/PG (prostaglandin) pathway might be involved, since PG are not only inflammation mediators, but also algogenic mediators [44]. Indeed Miladiyah et al. [45] demonstrated the analgesic activity of *Manihot esculenta* leaf extracts in vivo on mice. This effect was considered as being of pharmacological level with an estimated potency in the range of paracetamol. Considering our results on inflammation, one can hypothesize that *Manihot esculenta* leaf extracts may reduce nociception through their anti-inflammatory capacity, i.e., reduction of inflammatory mediator’s production known to be involved in pain signaling. Therefore, *Manihot esculenta* leaf extracts could behave like non-steroidal anti-inflammatory drugs such as indomethacin, for example. Further investigations are needed to reveal the precise pharmacology of the anti-inflammatory and immunomodulatory effects of cassava leaf extracts. Such insight associated to the study of the impact of bioavailability (i.e., absorption through gastrointestinal mucosa) on the relative composition of the extracts are necessary to better understand the potential ability of *Manihot esculenta* leaves to manage diseases with an inflammatory component. With a positive impact on in vivo acute inflammation being already reported [43], the possible beneficial nutritional and health effects of cassava leaves on chronic low-grade inflammation pathological conditions, i.e., metabolic diseases, remain to be explored. In such an endeavor, it will be necessary to consider food processing modalities and their influence on both nutritional, anti-nutritional, bioactive antioxidant and bioactive anti-inflammatory compounds qualities and relative quantities with regard to human health benefits. Indeed, cassava leaves contain anti-nutrients and toxic compounds that must be taken into consideration in order to valorize this vegetal matrix. Toxic molecules include cyanogenic glycosides (e.g., linamarin), cyanohydrin and free cyanides. Anti-nutritional factors are mainly represented by phytic acid, tannins and high fiber content. In order to optimize cassava nutrition and health value, the issue of leaf toxicity can be addressed by various appropriate and efficient detoxification processes to reach less than 10-ppm cyanides [46].

## 5. Conclusions

The results of our study clearly reveal that the phytochemical composition of cassava leaves is associated with interesting bioactive properties. Evaluation of the antioxidant activity of the three extracts of cassava leaves was comparable to rosemary extract. An inhibition of superoxide anion production on living cells was also noted in a dose-dependent manner. 

A significant dose-dependent inhibition of proinflammatory markers, including IL-6, TNF-α, MCP-1 and PGE-2, was also recorded on stimulated macrophages stimulated pretreated with different concentrations of cassava leaf extract.

These results clearly suggest that cassava leaves have antioxidant, anti-inflammatory and immunomodulatory activities that may contribute to the prophylaxis and/or reduction of the incidence of non-communicable metabolic diseases.

In this perspective, further research can be initiated on *Manihot esculenta* leaves to (1) explore their effects on preclinical models of metabolic syndrome with chronic low-grade inflammation, (2) uncover the combination of compounds, both at the qualitative and quantitative levels, that underlie their health benefits, and (3) define the different transformation processes that could optimize their nutritional and health qualities. Our research group is engaged in these investigations and preliminary results are encouraging.

## Figures and Tables

**Table 1:** *Manihot esculenta* composition in fibers, ash and carotenoids for the three samples from the Réunion (CL.R), Guinea (CL.G), Costa Rica (CL.Cr). Values are expressed as mean plus or minus SD (*n* = 3). Rosemary (R) is used as reference control in all test (a, b, c; *p* < 0.05).

Phytochemical Composition of Cassava Leaves (% DM/mg·100 g^−1^)						
Sample	Fibers			Ash	Carotenoids	
	Hemicellulose	Cellulose	Lignin		Lutein	β-Carotene
CL.R	12.32 ± 0.65 ^b^	7.59 ± 0.38 ^a^	22.34 ± 0.35 ^c^	7.62 ± 0.28 ^a^	26.1 ± 2.6 ^a^	35.1 ± 5.35 ^a^
CL.G	13.97 ± 0.22 ^a^	7.43 ± 0.36 ^a^	26.69 ± 0.40 ^a^	6.51 ± 0.49 ^b^	5.0 ± 0.3 ^b^	9.0 ± 0.47 ^c^
CL.Cr	12.46 ± 0.50 ^b^	8.33 ± 0.34 ^a^	20.61 ± 0.55 ^b^	7.55 ± 0.35 ^a^	23.7 ± 2.1 ^a^	25.6 ± 1.94 ^b^

Fiber and ash contents are expressed in %Dry Matter which is equivalent to g/100 g of samples. B-carotene content is expressed in mg/100 g of sample.

**Table 2:** *Manihot esculenta* antioxidant potential of the three samples from the Réunion (CL.R), Guinea (CL.G), Costa Rica (CL.Cr)**.** Values are expressed as mean plus or minus SD (*n* = 3) (*p* < 0.05). Rosemary (R) is used as reference control in all test (a, b, c; *p* < 0.05).

Antioxydant Activity				
Samples	TPC in mg GAE/g EDW	DPPH		ORAC µmol TE/g EDW
		µmol TE/g EDW	Inhibition (%) at 1 mg/mL	
CL.R	109.71 ± 5.48 ^b^	112.43 ± 8.61 ^b^	78.81 ± 5.37 ^a^	3233.82 ± 443.63 ^a^
CL.G	100.65 ± 11.30 ^b^	93.00 ± 13.41 ^b^	64.33 ± 3.47 ^b^	3083.16 ± 295.10 ^a^
CL.Cr	133.75 ± 4.21 ^a^	95.05 ± 1.49 ^b^	73.79 ± 4.88 ^ab^	2904.56 ± 180.26 ^a^
R	47.82 ± 2.44 ^c^	269.67 ± 14.55 ^a^	N. D	2624 ± 45.08 ^a^

TE (Trolox Equivalent), EDW (Equivalent Dry Weight) and GAE (Gallic Acid Equivalent).

## Data Availability

Not applicable.
